# Servant leadership and employee voice behavior: a cross-level investigation in China

**DOI:** 10.1186/s40064-016-3264-4

**Published:** 2016-09-17

**Authors:** Aimin Yan, Yigui Xiao

**Affiliations:** Business School of Central South University, Changsha, Hunan China

**Keywords:** Servant leadership, Psychological safety, Voice behavior, Supervisor-subordinate Guanxi, Civil servant

## Abstract

This study tested the influence and mechanisms of servant leadership on voice behavior, including the mediating role of psychological safety, and the moderating role of supervisor-subordinate Guanxi. Data were collected from 430 civil servants and their immediate supervisors in Changsha, China. Cross-Level investigation revealed that servant leadership had a significant influence on voice behavior, psychological safety mediated the relationship between servant leadership and voice behavior, while supervisor-subordinate Guanxi negatively moderated the relationship between servant leadership and voice behavior.

## Background

Times are changing and so are our views on leadership behavior, the leadership behavior of government administrators or entrepreneurs should evolve with the changing history、culture、economic and politics (Van Wart 2013). Since the building of service-oriented government concept was presented in 2004, 17th, 18th Communist Party of China National Congress and other congresses stepped this decision further, which raised severe leadership challenges for all levels of government especially the local government. The voice of “serve the people” surging, which asked all levels of officials go extra miles to turn to a servant leader (Han et al. 2010). As a unique new ethical leadership field of research for leadership scholars, servant leadership gradually became research focus of all walks of life (Liden 2012). Servant leadership as an understanding and practice of leadership that placed the good of those led over the self-interest of the leader, emphasizing leader behaviors that focused on follower development, and de-emphasizing glorification of the leader. It emphasized leaders’ moral behavior, protecting followers from self-interested leaders pursuing ends for their own selfish gain (Graham 1991). Servant leaders also recognized their moral responsibility to the success of the organization as well as to the success of their subordinates, the organization’s customers, and other stakeholders (Ehrhart 2004). One of a most vital criterions which measured a successful servant leader was whether the followers becoming a servant themselves or not (Greenleaf 1977). Consequently, such leaders imbued the importance of service within as well as outside of the organization.

Servant leadership served as an efficient leadership which not only owned unparalleled merits in forecasting the attitudes and behaviors of employees, but also perfectly matched the reform goal of building a service-oriented government (Wu et al. 2013). However, too much attentions were attached to the influence of abusive leadership, transformational leadership and paternalistic leadership to the voice behavior of subordinates, there is a need to research the mechanism of servant leadership exert to employees voice behavior in public management of government (Detert and Burris 2007). Research has yet to systematically consider the interactive effects of servant leadership on employee behaviors, such as voice behavior, which hindered the building and perfection of servant leadership theory and practical utilizing.

Scholars had studied the mechanism of servant leadership such as procedural justice (Ehrhart 2004), trust (Miao et al. 2014), organization performance (Barbuto and Wheeler 2006) and so on, however, the results were scattered that there was still a necessary to uncover the “black box”(Hunter et al. 2013). High quality decisions made by leaders needed the voice of their followers, China’s largest offering advice and suggestions activities were happened during the National People’s Congress and Chinese People’s Political Consultative Conference in March. Representatives all around the nation to voice for the Communist Party so as to build a service-oriented government and better the lives of their people. We focused this research mainly on junior civil servants because of its convenience but also its significance. Junior civil servants served as “street bureaucrats” who standing at the border between government and society who were designed to face and serve the people, they familiared more with the loopholes, so the leaders who could obtain half the work with double results if their followers voice energetically, which was meaningful for the building of service-oriented government. Differentiate with the west, most civil servants in China hold a pessimistic expectation with voice, the beautiful voice tradition and its effects were weakened now. Hence how to motivate junior civil servants voice were of great significance in improving the service level of local government even the building of “service-oriented government” who were deeply affected by Chinese culture traditions.

As for voice behavior, leaders were critical to the voice process (Detert and Burris 2007), on the other hand voice were discretionary, challenge-oriented, and potentially risky in nature compared with other extra-role behaviors. These characteristics spurred us to think deeply, under which circumstances will subordinates voice? It seemed that social exchange theory only partially explained the “extra-role” feature but “challenging, interpersonal risk”. Voice as a result which was the interaction of individual and environment (Detert and Edmondson 2011), individual psychological perception of organization climate safe or not which would impact the choice of individual voice behavior, thus in this research we studies psychological safety as mediator variable between servant leadership and voice behavior. Finally, take “renqing” society of China into consideration, “Guanxi” existed particularly notable in government, the existence “supervisor-subordinate Guanxi” may also affect the reciprocity intentions, we studied “supervisor-subordinate Guanxi” as a moderator between servant leadership and voice.

## Methods

### Data collection

We collected data for this study from primary cadres in Changsha, Hunan, China. With the assistance of the Organization Department of CCPC of the city, we distributed separate questionnaires to the subordinates and their immediate supervisors (town chief or general secretary). Supervisors assessed subordinates’ voice behavior while subordinates assessed supervisors’ servant leadership, psychological safety and supervisor-subordinate Guanxi.

In total, 869 questionnaires were completed on site and returned immediately. After deleting unmatched questionnaires (either only subordinate or only supervisor questionnaire) and carelessly completed questionnaires, we obtained 473 matched supervisor subordinate dyads questionnaires. We attained this high response rate because we adopted ‘response enhancing techniques’, such as incentives, advance notice and hand delivery (Anseel et al. 2010). Of the 430 subordinates, 97.8 % were male, 80 % were between 40 and 45 years old, 90 % were communists. Moreover, 60.5 % of subordinates had a bachelor diploma, 80.5 % of subordinates had working more than 10 years in town.

### Measures

All materials were presented in Chinese. Where translation was necessary (e.g. servant leadership, psychological safety, supervisor-subordinate gaunxi and voice behavior), Brislin’s (1980) recommended translations and back-translation procedures were followed. All scales were measured on a five-point scale (1 = strongly disagree, 5 = strongly agree).

Servant leadership was assessed by Ehrhart (2004). Sample items included ‘my department manager spends the time to form quality relationships with department employees’ and ‘my department manager creates a sense of community among department employees’. The internal consistency reliability of the scale in our study was 0.95.

Psychological safety was assessed by Edmondson (1999). Sample items included ‘If you make a mistake on this team, it is often held against you’ and ‘Members of this team are able to bring up problems and tough issues’. The internal consistency reliability of the scale in our study was 0.82.

Supervisor-subordinate Guanxi was assessed by Chen et al. (2009). Sample items included ‘My supervisor and I always share thoughts, opinions, and feelings toward work and life’ and ‘I feel easy and comfortable when I communicate with my supervisor.’ The internal consistency reliability of the scale in our study was 0.93.

Voice behavior was assessed by Liang et al. (2012). Sample items included ‘Proactively develop and make suggestions for issues that may influence the unit’ and ‘proactively suggest new projects which are beneficial to the work unit’. The internal consistency reliability of the scale in our study was 0.87, 0.86.

## Our models and assumptions

### Servant leadership and voice behavior

Voice behavior became a research hotspot with the rising research of organizational citizenship behavior (OCB), which was defined as proactively challenging the status quo and making constructive suggestions. There are three inherent characteristics of voice behavior: discretionary, challenge-oriented, and potentially risky (Van Dyne and Le Pine 1998). Speaking up may be potentially risk because they involved pointing out need for improvement in a policy to those who may have devised, be responsible for, or feel personally attached to the status quo. And the target mostly were their supervisors who had the authority to administer rewards and punishments, and this power over subordinates’ pay, promotions, and job assignments makes leaders’ actions highly salient as cues for behavior. Once followers felt that the potential costs of voice behavior outweighed the likely benefits (Milliken et al. 2003), this discretionary behavior was frequently withheld (Van Dyne et al. 2003).

Undoubtedly, leaders played a critical role to motivate employees to voice. Transformational leadership which would increase the voice behavior of employees, however abusive leadership restrained voice behavior. Servant leadership as an effective leadership which improved the employee job satisfaction greatly (Irving and Longbotham 2006) and OCB (Walumbwa et al. 2010). Servant leaders focused on the benefits of their followers, who deemed subordinates not as a tool in organization. They saw the leader position more a channel for them to help, support and assistant their followers. According to social exchange theory, when a servant leader put followers’ benefit first, offered various assistant for followers to success, provided timely help, fully communicated and reasonable empowered in the course of work etc. these kinds of behaviors which could seemed as an invest input for them to establish a nice social relationship with their followers. When employees were treated fairly and served sincerely by a leader they trust, they were likely to think about their relationship with the leader in terms of social exchange rather than economic exchange. One way to reciprocate for such treatment is to engage in constructive voice behavior innovation-driven organizational citizenship behavior (Walumbwa et al. 2012; Walumbwa and Schaubroeck 2009). According to reciprocity principle,when employees felt being served and valued by leaders, they will response in return as a payback for their leaders, hence voice behavior like other organization citizen behavior was deemed for employees as an effective return to leaders. On this basis, we predicted that servant leadership would promote voice behavior in work units.

#### **Hypothesis 1**

Servant leadership is positively related to voice behavior.

### The mediating role of Psychological safety

Psychological safety referred to shared beliefs among work unit members that it was safe for them to engage in interpersonal risk taking (Edmondson 1999). Psychological safety went beyond perceiving and experiencing high levels of interpersonal trust; it also described a work climate characterized by mutual respect, one in which people were comfortable expressing their differences. Individuals acted differently under various psychological status and circumstances, the changing environment which would affect the degree of input or express while working. In environments characterized by accepting different ideas existence, error tolerable, adventure sustaining which would strengthen the employees’ psychological safety, they were more willing to undertake innovative tasks and courage work (Baer and Frese 2003), individuals with a psychological safety sense perceived little risk to their own welfare in engaging in voice behavior. Things were different greatly when an individual felt unsafe, they would inclined to hold their opinions and thinking, reduced the helping intension (Edmondson 1999). Voice behavior possessed double attributes, which could be seen as “extra-role” behavior but also more easily be deemed as antitheses behavior. Voice behavior was associated with discomfort, labelled a negative public image or label which was potentially risky. Due to the high risk of voice behavior, employees were more likely to weigh the cost and benefit before voicing because of its target who owned the right to award and punish which would fasten or slowed their occupational promotion. It was undoubtedly that employees will evaluate whether safe or not to make decisions, psychological safety was long seemed as an important cognitive variable which affected voice behavior (Detert and Burris 2007).

As far as psychological safety’s antecedent variables concerned, leaders were pivotal for removing the constraints that often discouraged followers from expressing their concerns and other ideas (Edmondson 1999). Servant leadership behavior created a pervasive social context that positively affected employees’ attitudes and behavior. Servant leaders acted in the best interest of the follower, trusted employees, focused on followers’ individual growth and development and established a good relationship with followers. These unique leadership traits conveyed the faiths that their leaders did care about them but also respect them, the followers high acknowledged with the servant leaders, which would improving the trust and respect extent of both sides, hence the followers could feel less risk. Because they trusted that their leaders made their own decisions from their values and principles but outsider pressures. They would feel more relax while doing risk things like voice behavior even which may result in unfavorable consequences, they were not afraid of it because they believed that their servant leader would make a just verdict (Dasborough and Ashkanasy 2002). In short, servant leaders was helpful to remote the downside of voice behavior, improving the sense of psychological safety to accelerate voice behavior. Together, these arguments about the effects of servant leadership on psychological safety and employee voice and the linkage between psychological safety and employee voice suggested the following hypothesis:

#### **Hypothesis 2**

Psychological safety mediates between servant leadership and voice behavior.

### The moderating role of supervisor-subordinate Guanxi

Recent years have witnessed the acceptance of Guanxi by Western management scholars and practitioners as an important factor contributing to the effectiveness of managing Chinese staff (Hom and Xiao 2011). “Guanxi” was a concept which filled in the daily life of everywhere in China, affected every aspects of people (Lin and Ho 2010). People utilized differential interaction patterns according to relatively strong or weak tie. Because of the socialization of organization members, which reduced the innate factors like blood relationship “Guanxi” forming. There was not an explicit boundary between work and life in China compared with the West, “Guanxi” was developed more in doing their works. “Supervisor-subordinate Guanxi” was beyond workplace permeated into a more private friendship between both sides (Grant and Berry 2011), which contained emotional interact and responsibility recognition and so on (Law et al. 2000). Unlike Leader-Member Exchange, supervisor-subordinate Guanxi could be accumulated through non-work activities such as dinners, gift-giving and doing favors, which were important channels for information and resource exchanging (Chen et al. 2004). In general, s–s Guanxi in Chinese organizations, which contains more group cognition and social emotional elements went beyond the job relationship, was very important due to the high power distance typical of China’s hierarchical culture (Wei et al. 2010).

The quality of s–s Guanxi affected the judgement of voice behavior, recognition of voice behavior risk, which was definitely a vital element for decision making while working. The potential cost-benefit would weigh while a subordinate voicing to supervisors. Although the leaders would not deny or resist with these behaviors in public, they were reluctant to see their leadership was doubted by their followers. Therefore we often saw in real life that people remain silence though they knew the whole story. Things would be totally different if there was a high trust and respect interaction quality Guanxi between both sides, which meaned that they could communicate more with each other beyond working times friendly. This kind of Guanxi made the subordinate had more chances to deeply know the intentions and thoughts of their supervisor. Once that established that the employee was brave enough to voice and what he did wouldn’t cause the supervisors’ antipathy. As for the supervisors, they were willing to give the chance to the subordinate speak, listening and thinking patiently, which would deem as a challenger to their authority. Leaders would see these as a reflection of competence and loyalty when they had achieved good results, which would further strengthen the trust of subordinate, the relationships on both sides would be harmony more, in return subordinate could obtain more gaining, like more promotion chance and better job arrangement (Law et al. 2000), relational rewards and positive performance appraisal (Chen et al. 2008). The leaders would conduct positive attribution and forgive them even if they didn’t achieved desired results. Li et al. (2014) found that the more close ties with a leader was, the more voice behavior occurred. Subordinates could form a social exchange relationship in which individuals return the benefits they receive from the relationship. Subordinates are more likely to ‘pay back’ their supervisors by engaging in voice behaviors that are discretionary and less likely to be rewarded by the formal organization system.

If supervisors and subordinates developed a relationship which was based on contract relationship, on the one hand voice behavior would not easily brought to the attention of the supervisor, and would be even mistaken for challenging the authority of the supervisor. If these suggestions did not achieve the desired results, which could hardly to achieve understanding of the leader even resulted in a negative impression, these would of course reduce the voice behavior. Hence, we proposed that supervisor-subordinate guanxi moderates the relationship between servant leadership and voice behavior.

#### **Hypothesis 3**

Supervisor-subordinate Guanxi moderates between servant leadership and voice behavior.

## Results

### Preliminary analysis

Means, standard deviation, and bivariate correlations among study variables were reported in Table 1. The inter-item reliabilities as measured by Cronbach alpha of our measures are reasonable. Servant leadership were positively related to psychological safety, and voice behavior (r = 0.193, p < 0.01), (r = 0.121, p < 0.05). Supervisor-subordinate Guanxi positively moderates between servant leadership and voice behavior (r = 0.108, p < 0.05).

**Table 1:** Means, standard deviations, and correlations among the variables

Variable	Mean	SD	1	2	3	4	5	6	7	8
1	0.09	0.281								
2	2.85	1.526	−0.008							
3	0.76	0.429	0.057	0.091						
4	2.60	0.953	0.043	0.178***	0.045					
5	2.81	0.660	0.099*	−0.125**	−0.028	0.069				
6	4.272	0.222	0.024	0.007	0.100*	0.017	0.112*			
7	4.003	0.589	−0.082	0.030	−0.042	−0.050	0.042	0.193***		
8	3.918	0.567	−0.168***	−0.065	−0.127***	−0.091	−0.055	0.108*	0.113*	
9	4.272	0.392	−0.173***	−0.087	−0.097*	−0.094	−0.104*	0.121*	0.142**	0.494***

*1* gender, *2* age, *3* politics status, *4* tenure, *5* highest education, *6* servant leadership, *7* psychological safety, *8* supervisor-subordinate Guanxi, *9* voice behavior

* p < 0.05, ** p < 0.01, *** p < 0.001

### Measurement model

Overall measurement quality was assessed using CFA with AMOS. The results showed that the four-factor model (servant leadership, psychological safety, supervisor-subordinate Guanxi and voice behavior) was a better fit to the data (χ^2^/df = 2.031, IFI = 0.914, TLI = 0.908, CFI = 0.916, SRMR = 0.0648, RMSEA = 0.051) than other models. According to these statistics, the measures appear to exhibit acceptable values and validity.

### Hypothesis testing

Servant leadership was defined in group level, ICC (1), ICC (2), Rwg were utilized to tested to the reliability. Servant leadership ICC (1) = 0.21, ICC (2) = 0.92 which all exceed the standard value of 0.12, 0.5, the mean value and median of Rwg were 0.992, 0.996 separately which all exceed the standards 0.7.

Given the multilevel nature of the data, Hierarchical linear modeling (HLM) analysis with the software HLM6.06 was utilized to test our hypotheses. We first separately ran null models with no predictors but voice and psychological safety as the dependent variable to test the between-group variance in outcome variable. The test results showed signify- cant between-team variances in psychological safety (σ^2^ = 0.291, τ_00_ = 0.057) and voice behavior (σ^2^ = 0.15, τ_00_ = 0.008).

Hypothesis 1 proposes a positive relationship between servant leadership and voice behavior. As shown by the results of model 3 in Table 2, servant leadership was positively related to voice behavior (M3, γ = 0.35, p < 0. 001). Thus, Hypothesis 1 is supported. Hypothesis 2 suggests psychological safety mediates between servant leadership and voice behavior. As shown by the results of models in Table 2 that servant leadership is positively related to psychological safety (M4, γ = 0.11, p < 0.05 ), and servant leadership, psychological safety and voice behavior tested in the meantime (M5, γ = 0. 26, p < 0.05) showed that psychological safety is positively related to voice behavior, servant leadership was positively related to voice behavior (M6, γ = 0.33, p < 0.001). Hypothesis 2 is supported though the mediating effect need Sobel test. ${\text{z = ab}}/\sqrt {\mathop a\nolimits^{2} \mathop s\nolimits_{b}^{2} + \mathop b\nolimits^{2} \mathop s\nolimits_{a}^{2} }$, a = 0.11, b = 0.26, *s*_a_ = 0.05, *s*_*b*_ = 0.06, z = 1.96 > 0.97, Hypothesis 2 is supported.

**Table 2:** HLM results

Variable model	Psychological safety		Voice behavior				
	M1	M4	M2	M3	M5	M6	M7
Intercept	4.00***	3.53**	4.27***	3.06***	2.86***	2.97***	−8.46**
Level-1							
Gender		0.03		0.01	0.01	0.01	−0.11
Age		0.01		−0.01	−0.01	0.01	0.01
Politics status		−0.04		−0.07	−0.06	−0.07	−0.04
Tenure		−0.04		0.01	0.01	0.01	−0.02
Highest education		−0.10		−0.06	−0.06	−0.05	0.02
Psychological safety					0.26*		
SSGX							2.99***
Level-2							
Servant leadership		0.11*		0.35***		0.33***	
SSGX×SL							−0.67***
Variance decomposition							
σ^2^	0.291	0.291	0.146	0.146	0.145	0.271	0.139
τ_00_	0.057	0.061	0.008	0.009	0.055	0.016	0.014

Regression coefficients are robust standard errors of unstandardized coefficients

*SL* servant leadership, *SSGX* supervisor-subordinate Guanxi

* p < 0. 05, **p < 0. 01, *** p < 0.001

With respect to hypothesis 3 that supervisor-subordinate Guanxi moderates the relationship between servant leadership and voice behavior. As shown by the results of model 7 in Table 2, we test supervisor-subordinate Guanxi, servant leadership, voice behavior and the interaction of supervisor-subordinate Guanxi and servant leadership, servant leadership is positively related to voice behavior (γ = 2.99, p < 0.001), the interaction (γ = −0.69, p < 0.001), Hypothesis 3 is not supported.

## Discussion

This study was among the initial multi-level attempt to investigate the consequences of servant leadership on voice behavior in civil servants of public sectors. We found that servant leadership is positively related to voice behavior, psychological safety mediates between servant leadership and voice behavior while supervisor-subordinate Guanxi moderates negatively the relationship between servant leadership and voice behavior.

The results of this study have several important theoretical implications. Firstly, this research is echoed actively with Hunter et al. (2013), Parris and Peachey (2013) that more native researches to servant leadership. We developed an integrated model by introducing psychological safety and supervisor-subordinate Guanxi which provided a new perspective for government public administration voice behavior in cultures such as China that have high power distance unique traditions.

Secondly, Consistent with Burris (2012), we also found that psychological safety mediates the relationship between servant leadership and voice behavior in Chinese context. Then, this research successfully linked servant leadership and voice behavior together, revealed a psychological process that an ethical, people-oriented, service-oriented servant leader motivating followers to voice. Under the frame work of social exchange theory, on the foundation of reciprocity principal explaining that how a servant to develop a high quality of exchange. Voice behavior is a voluntary behavior which is risky because it start from challenging the existed institution or their immediate leader. Employees are hesitating to put constructive ideas forward because of the potential losing. Through building a sensation of belonging and attachment by servant leaders’ high serving ethical leadership (Zhu et al. 2013), the followers will pay their supervisors back through offering suggestions and advice, both sides can form a high quality of social exchange rather than economic exchange.

Interestingly, however, our data suggested that hypothesis 3 is not supported, supervisor-subordinate Guanxi moderates negatively the relationship between servant leadership and voice behavior. One of the reasons may the special civil servants respondents of China that the dark sides of supervisor-subordinate Guanxi worked more while reciprocity principal was confined because of its unique situation (Farh et al. 2007). Although the modernization of China achieved unsurpassed accomplishment, government power still need to further institutionalized, employees’ career development and promotion this kind of personnel decisions were affected by their supervisor mostly in government compared with other countries around the world (Chen et al. 2009), the supervisor-subordinate Guanxi developed in working process and invested after work was more a tool for employees to get benefits, which was an unhealthy interaction (Zhang et al. 2014). Hence, even both sides formed a good relationship, the first aim of employees was gain benefit rather than a better working condition or improving related to work, employees remain silence because of the potential lose, the best choice was remain silence to get an nice first impression from their supervisor so as to their own purpose. As stated by some Chinese proverbs, “Too much talk leads to error, careless talk makes trouble” and “Speech is silver, and silence is gold”. And harmony is precious. Higher collectivism tendency individual emphasized interior harmony much (Spreitzer et al. 2005), especially in government department. These civil servants were more easily be affected by these culture traits and traditions. They pursue stable than rather than competition, they will avoid contradiction as possible as they even tolerant unfair treat sometimes to get well along with each other not to mention high risk voice behavior even they had a good Guanxi with their supervisor.

Loyalty and obedience. The high power distance in Chinese government, civil servants were more easily used to act based on hierarchical difference (Hwang 2000). Supervisor was considered more a person but a symbol represented the organization and authority, they will accept all the orders and arrangements without any doubts even some of them were unreasonable to show their loyalty to win recognition from their supervisor.

Our research brings significant implications for practice. We have shown that servant leaders renders employees more likely to voice behaviors. This result ought to serve as a cue that servant leaders conforms to management requirements in era of democracy which should be encourage in government department. A wise leader should evolve and change their leadership style to motivate followers’ positive attitude and behaviors, so as to achieve higher performance simultaneously. Besides, leaders should take supervisor-subordinate Guanxi cautiously, avoiding the dark side of it, weaken the limitation of traditional values by sincere service, forgiveness and support which are based on high ethical equality to offset employees’ incorrect values.

As with any empirical study, ours has several limitations that point to avenues for future research. First, we did not empirically test the possible psychological mechanisms between servant leadership and voice behavior, because our theoretical model centers on psychological safety only. As Tangirala and Ramanujam (2008) found that personal control which affect voice behavior also, future research should put other variables to consideration for fully research. Second, servant leadership is a special leadership, which is overlapped with transformational leadership, ethical leadership and so on, future research should control these leaderships to gain a more reliable result.

## Conclusion

In summary, this study was among the initial multi-level attempt to investigate the consequences of servant leadership on voice behavior in civil servants of public sectors. Cross-Level investigation revealed that servant leadership had a significant influence on voice behavior, psychological safety mediated the relationship between servant leadership and voice behavior, while supervisor-subordinate Guanxi negatively moderated the relationship between servant leadership and voice behavior.

## References

[CR1] Anseel Frederik (2010). Response rates in organizational science, 1995–2008: a meta-analytic review and guidelines for survey researchers. J Bus Psychol.

[CR2] Baer M, Frese M (2003). Innovation is not enough: climates for initiative and psychological safety, process innovations, firm performance. J Org Beh.

[CR3] Barbuto JE, Wheeler DW (2006). Scale development and construct clarification of servant leadership. Group Org Manag.

[CR4] Brislin RW (1980) Translation and content analysis of oral and written material, in handbook of cross-cultural psychology. HC Triandis and JW Berry (editors) Boston: Allyn and Bacon, 1: 389–444

[CR5] Burris ER (2012). The risks and rewards of speaking up: managerial responses to employee voice. Acad Manag J.

[CR6] Chen C, Chen Y, Xin K (2004). Guanxi practices and trust in management: a procedural justice perspective. Org Sci.

[CR7] Chen Y, Friedman R, Yu E, Sun F (2008) Examining the positive and negative effects of Guanxi: a multi-level analysis of Guanxi and procedural justice. Paper presented at Meeting of 3th international association for chinese management research, Guangzhou, August

[CR8] Chen Y, Friedman R, Yu E, Fang WH, Lu XP (2009). Supervisor-subordinate Guanxi: developing a three-dimensional model and scale. Manag Org Rev.

[CR9] Dasborough MT, Ashkanasy NM (2002). Emotion and attribution of intentionality in leader–member relationships. Leadersh Quart.

[CR10] Detert JR, Burris ER (2007). Leadership behavior and employee voice: is the door really open?. Acad Manag J.

[CR11] Detert JR, Edmondson AC (2011). Implicit voice theories: taken-for-granted rules of self-censorship at work. Acad Manag J.

[CR12] Edmondson A (1999). Psychological safety and learning behavior in work teams. Adm Sci Quart.

[CR13] Ehrhart MG (2004). Leadership and procedural justice climate as antecedents of unit-level organizational citizenship behavior. Pers Psychol.

[CR14] Farh JL, Hackett RD, Liang J (2007). Individual-level cultural values as moderators of perceived organizational support- employee outcome relationships in china: comparing the effects of power distance and traditionality. Acad Manag J.

[CR15] Graham JW (1991). Servant leadership in organizations: inspirational and moral. Leadersh Quart.

[CR16] Grant AM, Berry JW (2011). The necessity of others is the mother of invention: intrinsic and prosocial motivations, perspective taking, and creativity. Acad Manag J.

[CR17] Greenleaf RK (1977). Servant leadership a journey into the nature of legitimate power and greatness.

[CR18] Han Y, Kakabadse NK, Kakabadse A (2010). Servant leadership in the People’s Republic of China: a case study of the public sector. J Manag Dev.

[CR19] Hom PW, Xiao Z (2011). Embedding social networks: how Guanxi ties reinforce chinese employees retention. Org Behav Hum Decis Process.

[CR20] Hunter EM, Neubert MJ, Perry SJ, Witt LA, Penney LM, Weinberger E (2013). Servant leaders inspire servant followers: antecedents and outcomes for employees and the organization. Leadersh Quart.

[CR21] Hwang KK (2000). Chinese relationalism: theoretical construction and methodological considerations. J Theory Soc Behav.

[CR22] Irving JA, Longbotham GJ (2006) Team effectiveness and six essential servant leadership themes: a regression based on items in the organizational leadership assessment [C], Servant Leadership Research Roundtable

[CR23] Law KS, Wong CS, Wang DX, Wang LH (2000). Effect of supervisor-subordinate Guanxi on supervisory decisions in China: an empirical investigation. Int J Hum Res Manag.

[CR24] Li JS, Wu LZ, Liu D, Kwan HK, Liu J (2014). Insider maintain voice: a psychological safety model of organizational politics. Asia Pac J Manag.

[CR25] Liang J, Farh CC, Farh JL (2012). Psychological antecedents of promotive and prohibitive voice: a two-wave examination. Acad Manag J.

[CR26] Liden RC (2012). Leadership research in Asia: a brief assessment and suggestions for the future. Asia Pac J Manag.

[CR27] Lin Lianghuang, Ho Yulin (2010). Guanxi and OCB: the Chinese cases. J Bus Eth.

[CR28] Miao Qing, Newman Alexander, Schwarz Gary, Lin Xu (2014). Servant leadership, trust, and the organizational commitment of public sector employees in China. Public Adm.

[CR29] Milliken FJ, Morrison EW, Hewlin PF (2003). An exploratory study of employee silence: issues that employees don’t communicate upward and why. J. Manag Stud.

[CR30] Parris DL, Peachey JW (2013). A systematic literature review of servant leadership theory in organizational contexts. J Bus Eth.

[CR31] Spreitzer GM, Perttula KH, Xin K (2005). Traditionality matters: an examination of the effectiveness of transformational leadership in the united states and taiwan. J Org Behav.

[CR32] Tangirala S, Ramanujam R (2008). Exploring nonlinearity in employee voice: the effects of personal control and organizational identification. Acad Manag J.

[CR33] Van Dyne L, Le Pine JA (1998). Helping and voice extra-role behaviors: evidence of construct and predictive validity. Acad Manag J.

[CR34] Van Dyne L, Ang S, Botero IC (2003). Conceptualizing employee silence and employee voice as multidimensional constructs. J Manag Stud.

[CR35] Van Wart M (2013). Administrative leadership theory: a reassessment after 10 years. Public Adm.

[CR36] Walumbwa FO, Schaubroeck J (2009). Leader personality traits and employee voice behavior: mediating roles of ethical leadership and work group psychological safety. J Appl Psychol.

[CR37] Walumbwa FO, Hartnell CA, Oke A (2010). Servant leadership, procedural justice climate, service climate, employee attitudes, and organizational citizenship behavior: a cross-level investigation. J Appl Psychol.

[CR38] Walumbwa FO, Morrison EW, Christensen A (2012). Ethical leadership and group in-role performance: the mediating roles of group conscientiousness and group voice. Leadersh Quart.

[CR39] Wei LQ, Liu J, Chen YY, Wu LZ (2010). Political skill, Guanxi with supervisor and the career development of subordinates: evidence from Chinese firms. J Manag Stud.

[CR40] Wu L, Tse EC, Fu P, Kwan HK, Liu J (2013). The impact of servant leadership on hotel employees “servant behavior”. Cornell Hosp Quart.

[CR41] Zhang L, Deng Y, Wang Q (2014). An exploratory study of chinese motives for building supervisor-subordinate Guanxi. J Bus Eth.

[CR42] Zhu W, Wang G, Zheng X (2013). Examining the role of personal identification with the leader in leadership effectiveness: a partial nomological network. Group Org Manag.

